# Supplementary material for: Innovation network structure, government R&D investment and regional innovation efficiency: Evidence from China
Source: PLoS One. 2023 May 22;18(5):e0286096. doi: 10.1371/journal.pone.0286096 (PMC10202290; doi:10.1371/journal.pone.0286096)
Supplement: Attachment — Submitted filename: Response to Reviewers.docx [file pone.0286096.s002.docx]

Thank you for your letter and for the reviewers' and editors' comments concerning our manuscript. These comments are very helpful for revising and improving our paper. We have studied comments carefully and have made corrections which we hope meet with approval.

**Reviewer #1 comments:**

This paper studys the relationship among innovation network, government RD investment and regional innovation efficiency including commercialization, the results sound right as dividing samples into four groups of A,B,C and D, and some deeper reasons are in want to explain why.

**Response:**

Thank you for your comment of this paper. Based on the panel data of 30 provinces in China from 2011 to 2019, this paper uses a two-stage DEA model to measure regional innovation efficiency, and uses non-parametric empirical method to test the impact of innovation network structure and government R&D input on regional innovation efficiency. The innovation of this paper is that on the basis of the original research, the research area is divided into groups A,B,C and D, hoping to give more targeted suggestions for different types of areas.

**Reviewer #2** **comments1:**

1.Introduction：

The introduction section provides a comprehensive overview of the connotation and influencing factors of innovation efficiency, pointing out the shortcomings of existing research and the contributions of this article. While the content is detailed, it also makes this section too lengthy. I recommend the author to add a DISCUSS chapter to expound the contributions and shortcomings of the article:

**Response:**

Thanks for your valuable comments and suggestions. We have revised the introduction. First, we simplified the description of the research status of innovation efficiency. Second, this paper first introduces the research status of innovation efficiency, and points out the shortcomings of existing research. Then we hope to supplement the existing research through this paper. There is a logical sequence, therefore, we did not delete the shortcomings of the existing research. Third, we moved the contribution to the end of the article and included a discussion section. Fourth, we selectively deleted meaningless sentences and paragraphs in the article. The details are as follows：

**Introduction**

……

Innovation efficiency is a key index to measure innovation achievements, which plays a very important role in decision-making [4]. Therefore, domestic and foreign scholars carry out research on the evaluation of regional innovation efficiency. However, many studies only focus on the efficiency evaluation from scientific and technological input to scientific and technological achievements output, and the evaluation on the transformation of scientific and technological achievements into economic benefits lacks depth [5]. Moreover, traditional research also ignores the fact that scientific research achievements may simultaneously play the roles of input and output in the innovation process, and at this time, the DMU will have an intermediate structure [6]. ~~For example, the index of patents commonly used in the literature today is not only the output result of R&D investment, but also the input factor of new product development. If we only investigate the R&D efficiency from the overall input and output, and do not explore the deep-seated causes of its inefficiency, we cannot solve the problem of how to improve the ineffective input. And the ultimate goal of regional innovation is to transform scientific and technological achievements into economic benefits [7].~~ In addition, although the existing research has explored the influencing factors of regional innovation efficiency in terms of economic development level [7], foreign direct investment [8], research and development subsidy [9], fiscal decentralization [10], human capital [11], financial development [12], industrial structure [13] and many other aspects, it has neglected that all kinds of factors play a role in the context of the innovation environment. Generally, enterprises have higher innovation willingness in a good innovation environment, while a bad innovation environment may cause the "innovation paradox" of high innovation demand and low innovation capability [14].

Innovation efficiency is mainly influenced by social network environment and policy environment. The social network environment emphasizes the influence of the relationship between innovation subjects on innovation efficiency. In order to obtain the innovation resources beyond the boundary of their own organization, the innovators establish stable and sustainable formal or informal links to form the innovation network structure [15]. ~~Some scholars construct the evaluation system of innovative network structure from the aspects of the number of network nodes and the strength of the relationship between nodes [17].~~The results show that the efficiency of regional innovation is directly affected by the structure of regional innovation network. Innovation networks in regions with high innovation efficiency generally have the following characteristics: a large number of innovation network components, high degree of network openness, smooth connections, and strong sense of cooperation [16]. ~~The more nodes, the more openness, the more unimpeded connections and the stronger sense of cooperation in innovation networks, the more beneficial to the improvement of regional innovation efficiency.~~ The policy environment emphasizes government support for innovation. The degree of government support is mainly measured by the intensity of government investment in R&D. Government R&D support is a policy tool to promote regional development, which is conducive to stimulating regional innovation potential [17]. The main means of government R&D support is the investment of R&D funds. Different types of government investment have different effects on innovation performance, but they all promote the improvement of regional innovation efficiency [18]. Data show that since the new century, China's R&D investment intensity continues to raise, from the scale of investment, China's total R&D funding now ranks second in the world.

It can be found that the existing studies have revealed the influence of innovation network structure and government R&D investment on innovation efficiency from different aspects, but there are still shortcomings: Firstly, the current researches on innovation network structure, government R&D investment and regional innovation efficiency mostly analyze innovation activities as a single process, and do not reveal the mechanism of each influencing factor on innovation in different development stages. Secondly, most studies only analyze the impact of innovation network structure or government R&D investment on innovation efficiency, and seldom pay attention to the compound effect of the two, which leads to the lack of deep understanding of innovation efficiency. In areas with underdeveloped innovation network structure, it is worth pondering whether the government can improve regional innovation efficiency by increasing R&D investment. To this end, this paper uses the two-stage DEA model to measure innovation efficiency at the R&D and commercialization stages in 30 Chinese provinces. This study verifies the combined effects of innovation network structure and government R&D investment on regional innovation efficiency with the help of nonparametric tests. Finally, we empirically analyze the results of the study, hoping to provide theoretical implications for innovation development in China.

~~The contribution of this paper is: First, we use the two-stage DEA to measure the efficiency of regional innovation, which has the advantage of dividing innovation activities into two stages: technological R&D and commercialization, the "Black Box" of the innovation system was broken by using the first stage of scientific and technological output as the input of the second stage. Secondly, we creatively analyze the innovation efficiency in the stage of technological R&D and commercialization from the perspective of innovation network structure and Government R&D investment, and pass the non-parametric test, to verify the difference of innovation efficiency in different stages of innovation activities between regions with different innovation network structure and government R&D investment, it is found that regions with backward innovation network structure can also stimulate the development of innovation by increasing government R&D investment. Thirdly, this paper proves that the combination of improving the innovation network structure and increasing the government R&D investment can make the regional innovation efficiency balanced, so as to provide guidance for the development of regional innovation differentiation in our country.~~

~~The rest of this paper is arranged as follows. In section 2, we review the literature and present our major hypotheses. In section 3, we introduce the data source, research method and index system of this paper. Section 4 and Section 5 respectively analyze the results of regional innovation efficiency and hypothesis testing. Finally, we will conclude with conclusions and an in-depth discussion of the findings, and then provide insights into innovation research and management practices.~~

Thanks!

**Reviewer #2comments2:**

2.Theoretical background and hypotheses：

In this section, the author elaborates on the article from four aspects: Regional innovation efficiency、Innovation network structure and regional innovation efficiency、Government R&D investment and regional innovation efficiency、Innovation network structure, government R&D investment and regional innovation efficiency,and propose reasonable assumptions separately. I think the hypotheses presented in this paper are well-formed and relevant to the research objectives. However, some of them need further elaboration to clarify their connections with the theoretical framework. Additionally, it would be helpful if you could clearly state the expected direction of each hypothesis (positive or negative) for easier interpretation.

**Response:**

Thanks for your valuable comments and suggestions. We think your comments are very constructive. While introducing the theoretical background, we focus on the theory itself but ignore to clarify their connections with the theoretical framework. The specific hypotheses don’t succinctly state whether the structure of innovation networks and government R&D investment have positive or negative effects on innovation efficiency. We have revised the theoretical background and hypotheses. First, we add a paragraph to the conclusion of the innovation network section and the government R&D investment section respectively. Second, we reformulate the specific assumptions. The details are as follows:

**Innovation network structure and regional innovation efficiency**

……

First, the innovation network structure has the characteristics of proximity [30]. On the one hand, the geographical proximity of innovation network structure promotes the collective action of enterprises, which in turn accelerates the knowledge flow between enterprises [31]. On the other hand, frequent face-to-face communication between enterprises close to each other deepens mutual trust, realizes resource sharing and improves innovation efficiency [32]. Geographic proximity also has a counter-effect, with excessive geographic proximity leading to spatial lock-in [33]. However, with the development of transportation and network, social proximity makes innovation gradually get rid of geographical constraints, and cross-regional cooperation among innovation subjects becomes possible. The social proximity of innovation network structure is increasingly considered as a key factor to promote knowledge flow and improve network performance [34]. Therefore, we believe that regions with developed innovation network structure have high R&D efficiency.

Second, the innovation network has realized the link to the innovation factor, reduced the innovation cost and accelerated the knowledge diffusion [15]. The "Optimality" of R&D personnel and the "Profit-seeking" of R&D Capital lead to the concentration of innovation elements in regions with more perfect innovation network structure, so as to form scale economy, and then promote regional innovation level [35]. Clusters with dense network structures have strong information transmission capabilities, and innovation agents can use innovation networks to obtain market information more easily and produce more market-oriented innovation outcomes [36]. The higher the density of innovation network is, the stronger the connection strength between network nodes is, which indicates that the motivation of collaborative innovation among innovation subjects is stronger, complementary information, knowledge resources, and so on, may be shared to a greater extent among heterogeneous innovators, contributing to the enhancement of innovation capacity and thus promoting the efficiency of regional knowledge transformation [27]. Based on the above analysis, we believe that regions with developed innovation network structure have high commercialization efficiency.

Taken together, the innovation network structure can have an important impact on regional innovation efficiency. The closer the constituent subjects of innovation network structure are to each other, the more conducive to the exchange of information and mutual learning of knowledge, and the output of more R&D results. The clustering of innovation factors can reduce innovation costs and thus increase the economic value of knowledge achievements. In general, proximity and low interaction costs increase the likelihood of achieving better innovation efficiency under the same conditions. The assumptions of this paper are therefore as follows:

**Hypothesis 1.** Innovation network structure affects regional innovation efficiency.

**Hypothesis 1a.** Innovation network structure has a positive promoting effect on R&D efficiency. **Hypothesis 1b.** Innovation network structure has a positive promoting effect on commercialization efficiency.

**Government R&D investment and regional innovation efficiency**

……

The first is that the technology spillover of R&D activities makes it impossible for enterprises to take all the returns of innovation factors, so the risk of market failure often occurs in the process of innovation investment [41]. As a special resource, government R&D investment provides financial support for enterprises' R&D activities, which effectively reduces the R&D cost and risk of enterprises in technological innovation [42]. When selecting R&D investment projects, the government will organize experts and professional evaluation bodies to select projects according to relevant regulations and regulations, thus ensuring the fairness of the evaluation process and the scientific nature of funded projects [43]. This makes government R&D investment can also be regarded as a credit endorsement, and enterprises can send positive financial signals to outside investors by obtaining government R&D investment with zero interest cost, thus enhancing their investment confidence [42]. Therefore, we believe that regions with high intensity of government R&D investment have high R&D efficiency.

Second, according to Samuelson's classical theory of public goods, it is inevitable that there will be inefficiency in government funding R&D activities with certain "public goods" attribute [40]. Moreover, when governments subsidize innovation, they tend to focus on the social benefits of innovation rather than on the direct economic benefits [37]. Government R&D investment is mostly applied to projects with long-term strategic significance, but this often makes it difficult to convert scientific research results into economic benefits [44]. At the same time, the attention of government leaders mainly focuses on the selection of projects and neglects the later supervision, so the efficiency of government R&D investment cannot be guaranteed [45]. When enterprises rely too much on government support, or innovation is mostly public institutions, market regulation is difficult to play a role. Based on the above analysis, we believe that regions with high intensity of government R&D investment have low commercialization efficiency.

Taken together, increasing the intensity of government R&D investment can reduce firms' R&D risks, improve R&D confidence, and increase R&D outcomes. However, the directionality of government R&D investment can weaken the role of the market in innovation and limit business innovation. The assumptions of this paper are therefore as follows:

**Hypothesis 2.** Government R&D investment affects regional innovation efficiency.

**Hypothesis 2a.** Government R&D investment has a positive promoting effect on R&D efficiency.

**Hypothesis 2b.** Government R&D investment has a negative inhibiting effect on commercialization efficiency.

**Innovation network structure, government R&D investment and regional innovation efficiency**

……The assumptions of this paper are therefore as follows:

**Hypothesis 3.** Innovation network structure and government R&D investment have a compound effect on regional innovation efficiency.

**Hypothesis 3a.** The R&D efficiency is relatively high in regions with underdeveloped innovation network structure but high government R&D investment intensity.

Thanks!

**Reviewer #2comments3:**

3.Methodology：

I appreciate the detailed descriptions of each indicator measurement process. However, I believe that some of these details are excessive and could be simplified to enhance readability without sacrificing information. The lengthy explanation for each indicator may cause unnecessary confusion for readers who are not familiar with the specific measurement process. As a result, I suggest that you consider revising this section to summarize the key steps in a more concise and straightforward manner.

Overall, please revise the indicators measurement section to balance thoroughness and clarity so that readers can easily follow your methodology without being overwhelmed by unnecessary detail.

**Response:**

Thanks for your valuable comments and suggestions. We have revised the methods section according to your suggestions. The specific changes and responses are as follows:

(i) On the measurement of innovation efficiency, we simplified the introduction of the index system and deleted some explanations of specific indicators. For the expressive aspect of DEA method, we simplified the interpretation of formulas so that readers can understand the article more easily. We hope to increase the readability of the article. The details are as follows:

**Measurement of innovation network structure**

There is no unified understanding on the selection of innovation efficiency index variables so far. Drawing on relevant literature research [14, 19, 27], this paper constructs an index system for innovation efficiency evaluation. The internal expenditure of R&D funds is selected to reflect the actual regional investment in R&D funds, and the full-time equivalent of R&D personnel is used to represent the investment in personnel of regional innovation efficiency. Intermediates are measured by invention patents and scientific papers. Per capita GDP and per capita disposable income can reflect the improvement of people's lives and the promotion of regional economic level by the achievements of scientific research. Sales revenue of new products can reflect the direct economic benefits brought by the progress of science and technology to the relevant units. The transaction contract amount of technology market reflects the level of technology achievements into market value. So we chose these four as the indicators of output in the commercialization stage.

Considering the complexity of innovation process, this paper uses a two-stage DEA model to measure innovation efficiency [14]. There are n decision-making units, and each decision-making unit has m kinds of input vector X and s kinds of output vector Y. For any decision-making unit, the model representation is shown in the following form (1) :

$\left\{\begin{matrix} \min\left(\theta-\varepsilon\left(\sum_{k=1}^{K} S^{-}+\sum_{l=1}^{L} S^{+}\right)\right) \\ s.t.\sum_{j=1}^{n} \lambda_{jk}X_{j}+S^{-}=\theta X_{k}^{j}\left(k=1,2,\cdots,K\right) \\ \sum_{j=1}^{n} \lambda_{jl}Y_{j}-S^{+}=Y_{l}^{j}\left(l=1,2,\cdots,L\right) \\ \sum_{j=1}^{n} \lambda_{j}=1 \\ \lambda_{j}\geq0,j=1,2,\cdots,n \\ S^{+}\geq0,S^{-}\geq0 \end{matrix}\right.$ （1）

In this model, $S^{+}, S^{-}$ represents input redundancy or output insufficiency of decision making unit. $\varepsilon$ means infinitesimal. $\theta$ is the efficiency evaluation value of the DMU. when $\theta=1$ and $S^{+}=S^{-}=0$, DEA is effective; if $\theta=1$ and $S^{+}\neq0$ or $S^{-}\neq0$, the DEA is weakly effective; if $\theta<0$, the DEA is invalid.

(ii) The rest of the changes are minor, with only some specific statement expressions being optimized. In these parts, we have simplified the expression in the original text. For example, entropy weight method and nonparametric test method don’t list detailed formulas. The index system of government R&D investment is put forward in the research method. The details are as follows:

……

According to the above four aspects, according to the principle of data availability and representativeness, the evaluation index system of innovation network structure is constructed (see Table 1). The maximum and minimum value method is used to deal with the data dimensionless, and the entropy weight method is used to calculate the index weights of all levels. Finally, the comprehensive value of the innovation network structure of each region is determined. The above mathematical formula of entropy weight method can be found in Liang Lina and Yu Bo [1615]. The length of the article is limited and will not be repeated here.

……

This paper introduces the government R&D investment intensity index. …… The formula is as follows:

O=$\frac{{RDE}_{kg}}{{RDE}_{k}}$∕$\frac{{RDE}_{g}}{RDE}$	(2)

O is the government R&D investment intensity index, RDE is R&D expenditure in the nation, RDE_K_ represents R&D expenditure in region, RDEg represents R&D expenditure of government, RDE_kg_ represents R&D expenditure of government in the region.

……

The steps of hypothesis testing are as follows: ① Kolmogorov-Smirnov test is performed on the above variables to test the normality of the data and determine the correctness of using nonparametric test. ② Because Mann-Whitney U test is often used to test whether there is a significant difference between two independent unpaired samples, we use Mann-Whitney U test to test hypothesis 1 and hypothesis 2. ③ Kruskal-Wallis H test is used when there are two or more samples, which is an extension of Mann-Whitney U test, so we use Kruskal-Wallis H test to verify hypothesis 3.The above nonparametric test statistics and their mathematical formulas are detailed in Conover [54], which will not be repeated here due to the limited length of the article.

Thanks!

**Reviewer #2comments4:**

4.Empirical part：

I found that the paper lacks a dedicated section for empirical analysis. For instance, the measurement of regional innovation efficiency and hypothesis testing can be combined to form an empirical analysis section, which is missing in the current manuscript. This section is essential for substantiating the arguments made in the earlier sections of the paper.

Therefore, I recommend that you revise your manuscript to include a comprehensive empirical analysis section.

**Response:**

Thank you for your valuable comments and suggestions. We have reconsidered the article and found your comments very constructive. Therefore, according to your suggestions, we combine the measurement of regional innovation efficiency with hypothesis testing to form the empirical analysis part.

**Reviewer #2comments5:**

5.Conclusion：

In this section,the authors have effectively utilized their hypotheses to substantiate their arguments, providing valuable insights that can be useful for academics and policymakers interested in this field. But I suggest that the author can provide a more macro policy suggestion from an overall perspective to improve the summary.

**Response:**

Thank you for your valuable comments and suggestions. We add more macro policy recommendations based on the conclusions of this paper. The details are as follows:

**Revelation**

The results of this paper provide some insights into how to improve innovation efficiency in different social network and policy environments.

In order to improve regional innovation efficiency, we need to build a sound innovation network structure. Expand the scale of the innovation network and form an innovation network mainly composed of enterprises, supplemented by universities, research institutions, governments, and technology intermediaries. We should make innovation networks more open, fully absorb foreign investment, enhance the sharing of innovation knowledge and achievements, and break down trade barriers. Strengthen the innovation network link and build the industry-university-research collaborative innovation mode, so as to improve the spontaneity, stability and sustainability of cooperation among innovation subjects. We should increase the number of structural holes in innovation networks, improve the construction of science and technology service platforms, promote the free flow of innovation information, and reduce the information asymmetry among innovation subjects.

In order to better leverage the government's role, we need to optimize the innovation policy environment and reasonably allocate government R&D investment. Actively play the government's functions in innovation strategy leadership, innovation environment construction and direct participation to lead the region to achieve innovation-driven development. Optimize the allocation of government R&D investment among innovation subjects. With the deepening of marketization, gradually cut the separate funding for research institutions and focus on supporting the industry-university-research collaborative innovation mode. We should explore a coordinated supervision and restraint mechanism for innovation investment subjects, so as to improve the transparency of the government's behavior in allocating innovation resources on the one hand and ensure the efficient use of government R&D investment on the other hand.

Thanks once more!

**Editors #comments1:**

Please review your reference list to ensure that it is complete and correct. If you have cited papers that have been retracted, please include the rationale for doing so in the manuscript text, or remove these references and replace them with relevant current references. Any changes to the reference list should be mentioned in the rebuttal letter that accompanies your revised manuscript. If you need to cite a retracted article, indicate the article’s retracted status in the References list and also include a citation and full reference for the retraction notice.

**Response:**

Thank you very much for your reminder. We have rechecked and corrected the references in the manuscript.
